# Towards an effective poliovirus laboratory containment strategy in Nigeria

**DOI:** 10.1186/s12889-018-6181-3

**Published:** 2018-12-13

**Authors:** Johnson Muluh Ticha, Kolawole Olatunji Matthew, Abdullahi Walla Hamisu, Braka Fiona, Pascal Mkanda, Peter Nsubuga, Eberto Tesfaye, Kehinde Craig, Etsano Andrew, Obi Emelife, Faisal Shuaib, Akinkugbe Folasade, Johnson Adeniji, Usman Adamu, Mohammed Dallatu, Geoffrey Oyeyinka, Holly Brown, Nwakasik Nnamah, Joseph Okwori, Chukwuike Chinedu, Ibikunle Anibijuwon, Adewumi Olubusuyi, Donbraye Emmanuel, Murtala Bagana, Marycelin Baba, Gumede Nicksy, Richard Banda, Sisay G. Tegegne, Ajiboye Oyetunji, Ousmane Diop, O. Tomori, Rui G. Vaz

**Affiliations:** 1 World Health Organization, Country Representative Office, Abuja, Nigeria; 2 World Health Organization, Regional Office for Africa, Brazzaville, Congo; 3 National Primary Health Care Development Agency, Abuja, Nigeria; 4 National Task Force on Polio Containment, Abuja, Nigeria; 5 University Teaching Hospital Polio Laboratory, Maiduguri, Nigeria; 6 Global Public Health Solutions, Atlanta, GA USA; 7 World Health Organization, Headquarters, Geneva, Switzerland; 8 Expert Review Committee on Polio and Routine Immunization, Abuja, Nigeria

**Keywords:** Laboratory, Containment, Poliovirus, Biosafety, Potentially infectious material, Survey, Inventory, Eradication

## Abstract

**Background:**

The Global Commission for the Certification of the Eradication of Poliomyelitis will declare the world free of wild poliovirus transmission when no wild virus has been found in at least 3 consecutive years, and all laboratories possessing wild poliovirus materials have adopted appropriate measures of containment. Nigeria has made progress towards poliomyelitis eradication with the latest reported WPV type 1 on 21 Aug 2016 after 2 years without any case. This milestone achievement was followed by an inventory of biomedical laboratories completed in November 2015 with the destruction of all identified infectious materials. This paper seeks to describe the poliovirus laboratory containment process in Nigeria on which an effective containment system has been built to minimize the risk of virus re-introduction into the population from the laboratories.

**Methods:**

A national survey of all biomedical facilities, as well as an inventory of laboratories from various sectors, was conducted from June–November 2015. National Task Force (NTF) members and staff working on polio administered an on-site questionnaire in each facility. Laboratory personnel were sensitized with all un-needed materials destroyed by autoclaving and incineration. All stakeholders were also sensitized to continue the destruction of such materials as a requirement for phase one activities.

**Results:**

A total of 20,638 biomedical facilities were surveyed with 9575 having laboratories. Thirty laboratories were found to contain poliovirus or potentially infectious materials. The 30 laboratories belonged to the ministries of health, education, defence and private organizations.

**Conclusions:**

This article is amongst the first in Africa that relates poliovirus laboratory containment in the context of the tOPV-bOPV switch in alignment with the Global Action Plan III. All identified infectious materials were destroyed and personnel trained to continue to destroy subsequent materials, a process that needs meticulous monitoring to mitigate the risk of poliovirus re-introduction to the population.

## Background

In May 1999, the World Health Assembly urged Member States to begin the process leading to laboratory containment of the wild poliovirus (WPV). On 25th May 2015, all World Health Organization (WHO) member countries endorsed the World Health Assembly resolution 68.3 on full implementation of the Polio Eradication and Endgame Strategic Plan 2013–2018 and with it the third Global Action Plan to minimize poliovirus facility-associated risk (GAP III) [1]. The Endgame Plan sets the goal of a polio-free world by 2018 [2]. One of the ways to achieving this is through the implementation of safe handling and containment measures for polioviruses to minimize the risks of the facility-associated reintroduction of the virus into the polio-free community [3]. In September 2015, the global commission for the certification of poliomyelitis (GCC) declared that wild poliovirus type 2 had been eradicated [4]. Polio outbreaks following certification might be caused by either wild or vaccine-derived poliovirus (VDPV), they could originate from various sources, and they could occur anywhere [5].

In 2014, Wolff et al. reported that containment phase 1 activities were completed in 154 (79%) of 194 WHO Member States, including all countries and areas of the polio-free regions and most polio-free countries in the remaining three regions [6]. This progress, particularly in several large industrialized countries, showed that wild poliovirus containment is operationally feasible [7].

The GCC will declare the world free of wild poliovirus transmission when no wild virus has been found in at least 3 consecutive years, and all laboratories possessing wild poliovirus materials have adopted appropriate measures of laboratory containment [8].

There is a paucity of data on the African continent or sub-region on the containment of WPV and potentially infectious materials. However, despite this, containment process was concluded in Zambia in 2012 with a survey of > 170 biomedical facilities ending up with one laboratory (i.e., the Lusaka University Teaching Hospital) having stocks of wild poliovirus and potentially infectious materials [9]. In 2008, in Sudan, 26 (6.1%) laboratories out of the 422 surveyed were found having poliovirus or potentially infectious materials [10]. By 2016 Nigeria had made progress towards poliomyelitis eradication with no case reported in 2 years, however between July and August 2016, four WPV1 cases were reported. These cases were restricted to Borno state in the northeast of the country which was facing security challenges.

This article seeks to showcase keys achievements of phase 1a poliovirus laboratory containment process in Nigeria, a foundation for an effective poliovirus containment system in a country without an essential facility.

## Methods

Phase 1a containment activities were carried out based on WHO guidelines.

### Creation of National Task Force (NTF) and work plan

Phase 1a containment activities commenced with the institution of the NTF in January 2015 by the Government. Six consultant virologists (one per geopolitical zone) were engaged from June–November 2015 for this activity. Both NTF and the consultants had a capacity building workshop in May 2015 (before the commencement of survey). The NTF elaborated a 6 months’ work plan (June–November 2015).

### National stakeholder’s sensitization

Apart from building the capacity of the NTF and consultants, a broad spectrum of stakeholders were sensitized in May 2015 on the concept and logistics of the national survey and inventory. Participants to the forum were drawn from several ministerial departments and government agencies amongst which were the National Agency for Food and Drug Administration and Control (NAFDAC) and the Medical Laboratory Council of Nigeria. Introductory letters on containment requirements were provided to stakeholders and copies were sent to various biomedical facilities for compliance with the process.

### Establishment of a preliminary list of biomedical facilities

At the commencement of survey in June 2015, a list of existing biomedical facilities in the country was provided by the Medical Laboratory Council of Nigeria, the Nigeria Medical Association, the Federal Ministries of Health, Defense and Agriculture.

### Pre-testing of survey questionnaire

The WHO generic questionnaire for poliovirus containment was adopted and pre-tested in one state of each of the six geo-political zones in May 2015 before the commencement of the survey in June 2015.

### Administration of questionnaires

The questionnaires were physically taken to all biomedical facilities in the country by the six consultants and supported by personnel working on the polio eradication activities. NTF members also conducted a survey of low-risk facilities during supervisory field visits.

Questionnaires were completed on site by the laboratory heads in the presence of the consultants. In rare occasions, the consultants had to come back some other time to collect the completed forms. An onsite inventory was carried out for facilities found with poliovirus infectious or potentially infectious materials.

These questionnaires were directed towards the identification of sample types, their sources, quantification, storage conditions including temperature, date of collection and methods of destruction once not more needed in the laboratory.

### Data management

Completed questionnaires for both survey and inventory were transmitted to WHO, and all the data were entered into an MS Access computer database for analysis and sharing with government and partners every week. All data generated from the study were checked manually for errors in filling responses, in some cases more information was requested, or the consultants returned to the facility for onsite review. Descriptive statistics such as mean, frequency, standard deviation, percentages and graphs were used to analyze the results. The data were analyzed using Epi Info version 3.5.3 (US Centers for Disease Control and Prevention) and the Statistical Program for the Social Sciences (SPSS 21) computer software packages.

### Supervision and coordination of containment process

Physical visits were made by the NTF members to supervise the containment process. They visited all laboratories in tertiary institutions as well as other laboratories identified with poliovirus or potentially infectious materials. Each NTF field visit was followed by meetings with the consultants to propose solutions to identified difficulties to the process.

### Laboratory inventory

The national inventory of laboratories having poliovirus infectious or potentially infectious materials was compiled indicating name of the laboratory, organization or ministerial department. The type of material identified, the quantity of material in store, storage temperature, the level of risk of the laboratory and the contact information of head of the facility was recorded.

#### Destruction of poliovirus infectious or potentially infectious materials

The identified poliovirus or potentially infectious materials in all biomedical facilities were destroyed using the best standard practices of autoclaving and incineration. The destruction was implemented through a directive from the Federal Government of Nigeria to all heads of the identified laboratories. The destruction process was supervised and monitored by NTF members.

#### Report of phase 1a activities

A report on the processes and findings of laboratory containment activities was written and forwarded to the Africa Regional Commission for the Certification of Poliomyelitis Eradication on 31st December 2015 through WHO country office as was required.

### Post-phase 1a laboratory containment activities

An enlarged stakeholder meeting (comprising mostly heads of the laboratories with an inventory of materials) was organized by the government. The meeting served as feedback on phase one containment activities as well as to train them on standard operating procedures (SOPs) for safe handling and destruction of subsequent polioviruses or potentially infectious materials in the laboratories henceforth. The forum was also charged to update the list of laboratories annually to be coordinated by the Medical Laboratory Council of Nigeria for which the NTF was to regularly visit and ensure compliance with biosafety particularly the immediate destruction of polioviruses or potentially infectious materials.

### Destruction of type 2 poliovirus after the trivalent Oral polio vaccine to bivalent Oral polio vaccine (tOPV to bOPV) switch

From 5th Feb 2016 to 5th July 2016, all type 2 poliovirus in both national polio laboratories in Ibadan and Maiduguri were destroyed through autoclaving and incineration.

## Results

Between June and November 2015, NTF conducted phase 1a poliovirus containment activities in all 36 states and the Federal Capital Territory. At the commencement of this survey in June 2015, a list of 16,722 biomedical facilities was provided by governmental and non-governmental institutions. At the end of the survey in November 2015, a total of 7340 (43.9%) of these 16, 722 facilities were seen to have closed down. Similarly, it was noted that 12,906 (77.1%) facilities were surveyed that were not on the initial list. Thus in November 2015, a total of 20,638 biomedical facilities were surveyed these including those on the initial list as well as new facilities discovered in the field. A total of 9575 (46.4%) of the 20,638 facilities surveyed had an inventory for poliovirus or potentially infectious materials (Table 1).

**Table 1:** Distribution of the number of biomedical facilities surveyed and identified laboratories by geopolitical zones June–November 2015

Geo-political zone	No of Facilities on initial official list	No of Facilities on initial official list found closed down	No of Facilities that were not on official list but surveyed	Total No of Facilities in the final list surveyed	No of Facilities with labs	No of Facilities without labs	Total number of labs surveyed
North Central	2452	1269	2852	4115	2512	1603	2512
North East	902	156	192	850	508	342	508
North West	1578	32	2126	3928	1044	2884	1044
South East	3223	1194	2085	3375	2327	1048	2327
South West	5610	2960	3159	4652	1777	2875	1777
South South	2957	1729	2492	3718	1407	2311	1407
Total	16,722	7340	12,906	20,638	9575	11,063	9575

A total of 4637 (48.6%) of 9575 laboratories surveyed were validated both by NTF and consultants. The response rate for the onsite administration of the questionnaires was 99% because 36 of the 9, 575 laboratories declined (initially) participation in the survey. A total of 30 (0.3%) of the 9575 laboratories surveyed were in possession of poliovirus infectious or potentially infectious materials. These 30 laboratories belonged to various Ministries as follows; Health 15 (50%), Education 11 (36.7%), Defence 1 (3.3%), Private Organizations 3 (10%). Most of these 30 laboratories (26.6%) were found concentrated in the North Central zone (Table 2) compared with the other five zones.

**Table 2:** Ministerial departments of laboratories found with poliovirus or potentially infectious materials during phase 1 containment activities June–November 2015

Geo-political zone	Number of laboratories with inventory	Ministerial Department of laboratories with inventory				Total
		Ministry of Health	Private Organization	Ministry of Education	Ministry of Defense	
North Central	8	7	0	0	1	8
North East	1	0	0	1	0	1
North West	6	3	0	3	0	6
South East	4	3	1	0	0	4
South West	6	1	1	4	0	6
South South	5	1	1	3	0	5
Total	30	15	3	11	1	30

A total of 189 (2%), 1946 (20.3%) and 7440 (77.3%) of the 9575 laboratories surveyed were classified as high, medium, and low risk respectively. These high-risk laboratories were mostly found in the South-East zone of the country (67/189, or 35.4%). A total of 6 (20%) of the 30 laboratories with inventory had Biosafety Level-2 (BSL-2) standards. These included the National Influenza Laboratory in Abuja, the WHO national polio laboratories in Ibadan and Maiduguri, Lagos University Teaching Hospital, University of Nigeria Enugu (bacteriology unit) and the University of Nigeria, Enugu (Nigeria Centre for Disease Control) (Table 3).

**Table 3:** Types and quantities of various polioviruses and or potentially infectious materials identified in 30 laboratories during inventory from June–November 2015 in Nigeria

Name of Laboratories	Type of sample	Quantity	Period of Collection	Storage Temperature(°C)
Modesty Med. Laboratory, Owerri, Imo	Stool	5	20/04/15	2
^a^N.C.D.C. Reference Laboratory, Enugu	Stool	2226	2010–2015	−60
^a^Bacteriology Unit, University of Nigeria Teaching Hospital Enugu	Cerebrospinal fluid	400	01/01/12	−60
IMSUTH Microbiology Lab - Orlu, IMO	Stool	8	09/01/15	2 to 8
^a^Central Research Laboratory, College of Medicine, Lagos University Teaching Hospital, Idi-Araba, LAGOS	Stool	110	2011–2012	− 20
N C D C Molecular Laboratory, Amino Kano Teaching Hospital, Kano	Stool	100	19/06/15	−60
Public health &Diagnostic Institute North West University, KANO	Stool	20	10/02/15	−32
Medical Microbiology Ahmadu Bello University Teaching Hospital Shika Zaria, Kaduna	Stool	5	10/06/15	− 20
Medical Department Barau Dikko Teaching Hospital Kaduna, KADUNA	Sera	1	17/06/15	−20
University Health Services, ABU Zaria, Kaduna	Stool	10	01/02/15	2 to 8
Garki Hospital Abuja Laboratory, FCT	Blood	2	2015	−20
Asokoro District Hospital Laboratory, FCT	Blood	4	2015	−20
Defense Headquarters Medical Centre Laboratory, Abuja, FCT	Sera	100	2015	room temperature
Micro-Biology Laboratory National Hospital, Abuja, FCT	Stool	14	2015	2
Measles / Yellow Fever Laboratory, Abuja, FCT	Sera	100	2015	−20
^a^National Influenza Reference Laboratory, Asokoro District Hospital, Abuja, FCT	Throat swabs	7894	2012	− 20 and − 70
University Of Abuja Teaching Hospital Laboratory, FCT	Sera	5	2015	−20
Rotavirus Laboratory, University of Ilorin, Kwara	stool	297	2013	−80
Landers Specialist Medical Laboratory, Rivers	Stool	1	2015	2 to 8
Medical Laboratory Services, University of Benin Teaching Hospital, EDO	Cerebrospinal fluid	41	2015	−80
Microbiology Laboratory, University of Port Harcourt) RIVERS	Water	7	Mar-Jul 2015	2 to 8
Research Laboratory, Plant Science & Biotechnology Department, University of Port Harcourt, Rivers	Water	7	2015	2 to 8
Medical Microbiology Laboratory, RIVERS	Stool	50	2015	−20
^a^WHO National Polio Laboratory, University of Maiduguri Teaching Hospital, BORNO	Poliovirus Isolates -	211		−20
Highland Specialist Hospital, Oyo	Throat Swap	13	June 2015	4
Drug Research Unit, Institute for Advanced Medical Research Ibadan, Oyo	Stool	928	June 2015	− 86
Ibarapa Community &Primary Healthcare Program Laboratory, Oyo	Stool	1	April 2013	4
Medical Microbiology, University College Hospital, Ibadan Oyo	Stool	400	Feb 2014	−20
^a^WHO National Polio Laboratory, University of Ibadan Teaching Hospital, OYO	Stool Stool suspensions Non-Polio Enteroviruses Sewage samples Un-typed Sewage samples Poliovirus isolates Wild Poliovirus Wild Poliovirus	6616 5221 5104 1126 400 1140 26 1978 730	2015 2015 2011–2014 2011–2014 2011–2014 2011–2014 2011–2015 2005–2014 Jan-Jul 2015	−20 − 20 − 20 −40 − 20 − 20 − 20 − 20 − 20
Bacterial Zoonosis, Kaduna	Stool	500	2015	−20

^a^Laboratories with BSL-2 facilities

Among the infectious materials found in the laboratories; were 11,291 stools samples located in 16 laboratories, 15,801 throat swab samples seen in 3 laboratories and 5221 stool suspensions in 1 laboratory.

The two most common samples found in the laboratories were stool (in 60%) and blood (in 16.7%) of laboratories. All of these infectious materials were destroyed during the survey under the supervision of the NTF members and consultants (Table 3).

As per WHO guidelines on containment, the 4th of February 2016 was the dateline set for all countries in the Africa region to destroy all poliovirus or potentially infectious materials that accumulated after the ones destroyed during phase 1a exercise. Thus, 646 VDPV2 and 3256 Sabin 2 from AFP stools were destroyed in both Ibadan and Maiduguri polio laboratories. Secondly, 775 mixtures of Sabin2 and VDPV2 from sewage water were destroyed in Ibadan laboratory on the same day. (Table 4).

**Table 4:** Quantities of Type 2 polioviruses destroyed on 4th February 2016 in the national polio laboratories

		Quantity destroyed in laboratories		
Type 2 poliovirus	Source	Ibadan	Maiduguri	Total
Sabin 2	Sewage samples	641	0	641
Sabin 2	AFP stools	1750	1506	3256
VDPV2	Sewage samples	135	0	135
VDPV2	AFP stools	181	465	646
Sabin 2 + VDPV2	Sewage samples	775	0	775

In alignment with the WHO containment guidelines, Nigeria had to continue to destroy all poliovirus or potentially infectious materials or transfer them to an essential facility with BSL-3 for polio. Nigeria has no such essential facility and thus the only option was to destroy it’s infectious or potentially infectious materials (Tables 4 and 5).

**Table 5:** Quantity of type 2 poliovirus destroyed between 5th February and 5th June 2016 in national polio laboratories

Polio lab	From AFP		From ES	
	VDPV2	Sabin 2	VDPV2	Sabin2
Maiduguri	2	153	N/A^a^	N/A^a^
Ibadan	0	29	1	51

^a^laboratory doesn’t test environmental samples. ES: Environmental sample

## Discussion

We found that 30 (0.3%) of laboratories in Nigeria had poliovirus or potentially infectious materials. Six (20%) of the 30 laboratories had biosafety level-2 standards for polio. In 2004, Sneyers et al. in Belgium found poliovirus or potentially infectious materials in 8 (1.9%) of the 411 facilities surveyed and all of them (100%) had BSL-2 [11]. Mpabalwamni et al. in 2012 reported that of the 170 biomedical facilities surveyed in Zambia, 24 (14.1%) were found having both wild polioviruses as well as potentially infectious materials. Our findings are in accordance with similar studies; Wolff et al. in 2014 surveyed 227,209 biomedical facilities in 154 countries of the 194 WHO Member States and 532 (0.2%) and identified 45 countries as retaining WPV or potentially infectious materials. These findings in Nigeria are again in agreement with those of Wolff et al. in which universities constituted the highest percentage of institutions retaining poliovirus materials. In Nigeria, all the poliovirus infectious or potentially infectious materials were all destroyed through autoclaving and incineration, this method was ideal to complete the phase 1a containment exercise since there wasn’t an essential facility, atleast a BSL-3 Laboratory in-country or elsewhere in Africa by then.

We noted that apart from various types of polioviruses, the most common potentially infectious materials were stool samples (11,291), throat samples (15,801) and sera (200). As per GAPIII these materials were destroyed through autoclaving and incineration in various laboratories. Appropriate biosafety measures are crucial for the prevention of poliovirus infection of laboratory workers and subsequent transmission to the community. Destruction of these materials have also been reported by several authors in Sudan and Zambia.

We also found out that the two WHO-accredited polio laboratories in the universities of Ibadan and Maiduguri in addition to potentially infectious materials had large quantities of type 1 and type 3 wild poliovirus as well as type 2 polioviruses (Sabin2 and VDPV2) from both sewage and AFP samples. These type 2 polioviruses were destroyed as per GAPIII requirements for Phase 1a activities and in the context of the trivalent OPV to bivalent OPV switch that was effective on 18th April 2016 in Nigeria. WHO supported labs thus constitute a potential source of poliovirus and potentially infectious materials in Nigeria and elsewhere as cited in the Zambia study reported by Mpabalwani et al. in 2012 where large quantities were identified in the Lusuka WHO supported polio laboratory. Environmental surveillance will play a critical role in monitoring the effectiveness of the switch [12]. In this regards in New Zealand, Q Sue et al. reported that OPV strains did not persist for extended periods after a vaccine switch [13]. Environmental surveillance is now being conducted in 18 states in Nigeria thus keeping the country in a good position to be able to monitor the circulation of any Sabin 2.

We also found that the Phase 1a laboratory survey offered the opportunity to establish a national list of 20,637 biomedical facilities as against 16,722 on the existing list. This updated list of biomedical facilities would serve as a useful baseline dataset for broader public health interventions beyond polio. This list of 20, 637 biomedical facilities was used during the April 2016 tOPV-bOPV switch in the country. Similarly in the Region of the Americas; six countries Argentina, Brazil, Chile, Colombia, Mexico and Peru established national registries that could be used beyond the goal of poliovirus containment. In Belgium, a list of 411 institutions was established for the survey with a respondent rate of 97.3% compared to the 99% rate in Nigeria. Nigeria conducted its survey using an on-site completion of the questionnaires contrarily to that in Belgium where in questionnaires were posted to various institutions. In Japan between 2004 and 2005, a total of 12,142 facilities were surveyed by the Ministry of Health, Labor and Welfare with a return rate of 99% [14]. This large number of laboratories surveyed in Nigeria is comparable with that in many other countries.

We also found out that 2, 20.3 and 77.3% of the laboratories with poliovirus infectious or potentially infectious materials were classified as high, medium and low risk respectively for the presence of poliovirus.

We recognize limitations of our study. It cannot be stated with certainty that all biomedical facilities were included in the survey in a large nation like Nigeria. Secondly, even if all facilities were included, they might not have been correctly identified to quantify the type and amount of poliovirus or potentially infectious materials. Despite these limitations, our study was able to reach a high number of laboratories resulting in the destruction of both potentially infectious materials and type 2 polioviruses for which the destruction continues systematically in all laboratories as such ensuring appropriate poliovirus containment thus reducing the risk of reintroduction of poliovirus into the population.

## Conclusions

The absolute laboratory containment of polioviruses or any virus, cannot be guaranteed, but a wealth of experience indicates that effective containment is technically and operationally feasible [15]. Phase 1a containment activities, have been achieved in Nigeria and ended with the destruction of all potentially infectious materials and type 2 poliovirus in all identified facilities. Laboratory staff capacity was strengthened, and the destruction process became systematic especially after the tOPV2-bOPV2 switch. This process is dynamics and needs meticulous monitoring to mitigate the risk of poliovirus leaving the laboratory back into the population. With few exceptions, the most extensive collections of potentially infectious materials are located in the research laboratories of a small number of developed and developing countries [16] with implications for a continuous survey and destruction of these materials. We recommend the regular capacity building of laboratory personnel on polio laboratory containment coupled with regular NTF visits to ensure strict adherence to Global Action Plan requirements as Nigeria prepares for polio-free status in a few years to come as well as the entire Africa region.

## Abbreviations

AFP: Acute Flaccid Paralysis
ARCC: Africa Regional Commission for the Certification of Poliomyelitis Eradication
bOPV2: Bivalent oral polio vaccine type 2
BSL-2: Biosafety Level 2
ES: Environmental Surveillance
GAPIII: Global Action Plan III
GCC: Global Commission for the Certification of poliomyelitis eradication
MS: Microsoft
NCDC: Nigeria Centre for Disease Control
NTF: National Task Force for poliovirus containment
SOP: Standard Operating Procedure
SPSS: Statistical Program for the Social Sciences
tOPV2: Trivalent oral polio vaccine type 2
USA: United States of America
VDPV2: Vaccine Derived Poliovirus type 2
WHO: World Health Organization
WPV: Wild Polio Virus
WPV1: Wild Polio Virus type 1
